# Comparative Analysis of Immunoactivation by Nanosecond Pulsed Electric Fields and PD-1 Blockade in Murine Hepatocellular Carcinoma

**DOI:** 10.1155/2020/9582731

**Published:** 2020-08-01

**Authors:** Maiweilidan Yimingjiang, Talaiti Tuergan, Xinhua Chen, Hao Wen, Yingmei Shao, RuiQing Zhang, Kasimu Aihaiti, Jing Xue, Tuerganaili Aji, Wei Zhang

**Affiliations:** ^1^Department of Pathology, The First Affiliated Hospital of Xinjiang Medical University, Urumqi, Xinjiang 830054, China; ^2^Department of Hepatic Hydatid and Hepatobiliary Surgery, Digestive and Vascular Surgery Centre, The First Affiliated Hospital of Xinjiang Medical University, Urumqi, Xinjiang 830054, China; ^3^Collaborative Innovation Center for Diagnosis and Treatment of Infectious Diseases, Key Laboratory of Combined Multi-organ Transplantation, Ministry of Public Health, Department of Hepatobiliary and Pancreatic Surgery, The First Affiliated Hospital, Zhejiang University, Hangzhou 310003, China; ^4^State Key Laboratory of Pathogenesis, Prevention, Treatment of High Incidence Diseases in Central Asia, Xinjiang Medical University, Clinical Medicine Institute, The First Affiliated Hospital of Xinjiang Medical University, Urumqi, Xinjiang 830054, China; ^5^Xinjiang Medical University, Urumqi, Xinjiang 830011, China

## Abstract

Nanosecond pulsed electric field (NsPEF) ablation effectively eliminates early-stage hepatocellular carcinoma (HCC) by local ablation and advanced HCC by inducing a remarkable and sustained host immune response. However, this approach is not sufficient to prevent cancer progression, and complementary approaches are necessary for effective immunotherapy. In this study, we evaluated the immunoactivating effects and mechanisms of action of nsPEF ablation and PD-1 blockade on an HCC orthotopic xenograft mouse model. Briefly, 24 C57BL-6J tumor-bearing mice were randomly assigned to three groups: nsPEF ablation group, anti-PD-1 administration group, and untreated control group. Tumor-infiltrating T, B, and NK cell levels and plasma concentrations of Th1 (IL-2, IFN-*γ*, and TNF-*α*), Th2 (IL-4, IL-5, IL-6, and IL-10), Th9 (IL-9), and Th17 (IL-17A, IL-17F, IL-21, and IL-22) cytokines were evaluated. Both nsPEF ablation and anti-PD-1 treatment induced immune cell infiltration in local tumors and modulated cytokine levels in the peripheral blood, with distinct changes in the two treatment groups. Based on these findings, both nsPEF ablation and PD-1 antibody administration can trigger a local and systemic immune response in a partially complementary manner, and nsPEF ablation should be considered along with PD-1 blockade for the treatment of HCC.

## 1. Introduction

Hepatocellular carcinoma (HCC) is one of the leading causes of cancer-related deaths worldwide and is a major concern owing to its increasing incidence. Because symptoms occur at late stages, early diagnosis is difficult and the prognosis is usually poor [1]. For the treatment of HCC, resection and transplantation are the main options; however, this is not often feasible in clinical settings, depending on the tumor size and underlying diseases, such as cirrhosis or other functional disorders [2]. A number of alternative new technologies have been developed, such as locoregional therapy, transcatheter arterial chemo-embolization, chemotherapy, biotherapy, and a single approved drug (sorafenib), and combinations of these approaches have been evaluated for unresectable HCC [3]. Inflammation (e.g., induced by viral hepatitis) progresses to cirrhosis and subsequently to HCC. Therefore, immunotherapy is a promising strategy [4].

Nanosecond pulsed electric field (nsPEF) is a high-power, minimally invasive locoregional ablation method based on high voltage technology [5]. As a novel nonthermal approach, nsPEF can ablate different solid tumors, including HCC [6–8], with ultrashort pulses, causing tumor cell apoptosis via multiple mechanisms [9, 10]. It also enhances macrophage [7] and T cell infiltration [11], consequently inducing an immuno-protective effect that defends against recurrence [12] as well as lung and bone metastases [8, 13, 14]. However, the efficacy of this method requires further investigation, and complementary approaches are necessary for effective immunotherapy in HCC.

Antibodies targeting the PD-1/PD-L1 axis have recently been investigated in phase 2 or 3 trials and have been approved for the treatment of various cancers [15]. However, a subset of patients who lack of PD-L1 expression do not benefit from anti-PD-1 therapy [16]. Furthermore, the prediction of responses to these antibodies is not reliable in HCC. Therefore, the rational combination of multiple methods able to induce antitumor immune responses may increase the number of patients with cancer who benefit from this approach. The combination of radiofrequency ablation (RFA) and PD-1 blockade synergistically enhances antitumor immunity in preclinical and clinical studies [17, 18].

In this study, we investigated the immunoactivating effects of both nsPEF ablation and PD-1 blockade on an HCC orthotopic xenograft mouse model. We compared the effects of both methods on the tumor microenvironment (TME) and peripheral blood to determine whether they have complementary effects and to obtain further insights into the immunoactivating effects of nsPEF and combination immunotherapy for HCC.

## 2. Materials and Methods

### 2.1. Cell Lines and Cell Culture

Human HCC cell lines Hepa1–6 were kindly provided by Professor Xinhua Chen of Zhejiang University, incubated at 37°C in a 5% CO_2_ incubator and cultured in DMEM supplemented with 10% FBS (Gibco, Carlsbad, CA, USA). The cell lines were mycoplasma-negative.

### 2.2. Animal Models

C57BL-6J mice were purchased from the Xinjiang Medical University at 6–12 weeks of age. After anesthesia, an abdominal incision of 1 cm was made, and orthotopic tumors were established by the injection of 1 × 10^6^ Hepa1–6 tumor cells under the capsule of the left liver lobe. This animal experiment was examined and approved by the laboratory animal welfare ethics committee of the Xinjiang Medical University.

### 2.3. Study Design and Treatment

**Figure 1:**
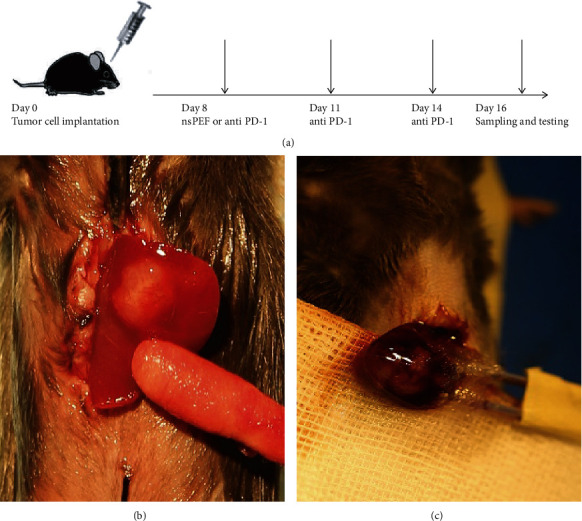
Establishment of an orthotopic HCC model and exposure to different therapeutic procedures. (a) Schematic drawing of the study design. Mice implanted with tumor cells were randomized into 3 groups; tumors were exposed to a single nsPEF ablation, and anti-PD-1 monoclonal antibodies were administered i.p. to mice every 3 days for a total of 3 times on day 8 after tumor implantation. On day 16, mice were sacrificed, and tumor and blood samples were collected for immune cell and cytokines detection. (b) Tumor growth on day 8 after implantation. (c) nsPEF ablation was performed with optimized parameters (300 ns, 20 kV/cm, 1000 pulses, 4 Hz).

Twenty-four C57BL-6J tumor-bearing mice were randomly assigned to three groups: nsPEF ablation group (*n* = 8), PD-1 antibody administration group (*n* = 8), and control group without any treatment (*n* = 8). NsPEF ablation was performed using self-made electrode needles on day 8 after tumor implantation, when the tumor size reached 8–10 mm in diameter. The distance between the needles was 0.5 cm. Tumors were exposed to a single nsPEF ablation with optimized parameters (300 ns, 20 kV/cm, 1000 pulses, 4 Hz). The PD-1 antibody was administered at 200 *μ*g (clone: J43; BioXCell, Lebanon, NH, USA) by an intraperitoneal injection every 3 days for a total of 3 times, beginning on day 8 after tumor implantation. On day 8 after treatment, blood samples were obtained by excising the eyeball immediately after euthanization, and serum was prepared by centrifugation (Figure 1). Tumor tissues were minced and digested with collagenase and hyaluronidase solution to prepare the single-cell suspension, which was filtered through a cell mesh and resuspended in Hank's media plus 1% fetal calf serum for further analyses.

### 2.4. Flow Cytometric Analysis and Cytokine Detection

Mouse-specific antibodies to T lymphocytes (CD45-PC7-A, CD3-PECY5, CD4-FITC, and CD8-PE), B lymphocytes (CD3-PECY5 and CD19 FITC-A), and NK cells (NK1.1-PECY7) were purchased from eBioscience (San Diego, CA, USA). A flow cytometric analysis was performed using a FACS flow cytometer DxFLEX (Beckman, Brea, CA, USA). Blood serum cytokine detection was performed using a mouse-specific cytokine kit (LEGENDplex Mouse Th Cytokine Panel (13-plex); Biolegend), according to the manufacturer's instructions. The assay sensitivity was pg/ml. All data were analyzed using Kaluza.

### 2.5. Statistical Analysis

Statistical analyses were performed using SPSS 23.0 and GraphPad Prism 7 (Version 7.00, GraphPad Software, Inc., La Jolla, CA, USA). Student's *t*-tests and one-way or two-way ANOVA were used to compare values between groups, and data are shown as means ± standard deviation. *P* < 0.05 was considered statistically significant.

## 3. Results

### 3.1. NsPEF Ablation and PD-1 Antibody Administration Significantly Enhanced T Cell-Mediated Antitumor Immunity by Different Mechanisms

**Figure 2:**
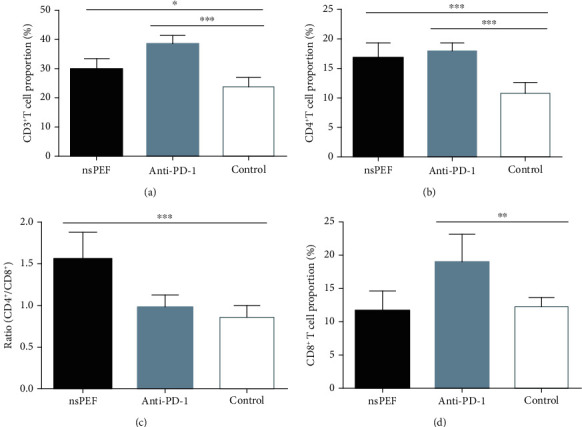
NsPEF ablation and anti-PD-1 therapy increased the infiltration of T lymphocytes into the tumor. (a) Percentages of CD3^+^ T cells within a single-cell suspension of tumor tissues. (b) Percentages of CD4^+^ T cells. (c) CD4^+^ : CD8^+^ ratio. (d) Percentages of CD8^+^ T cells (values represent means ± SED, ^∗^*P* < 0.05, ^∗∗^*P* < 0.01, ^∗∗∗^*P* < 0.001).

A flow cytometric analysis showed that the percentages of T lymphocytes were higher in both the nsPEF (*P* < 0.05) and anti-PD-1 groups (*P* < 0.001) than in the control group. CD4^+^ T cell proportions were also significantly higher in the two treatment groups than in the control group (*P* < 0.001), the frequency of CD8^+^ infiltrating T cells increased more highly in the anti-PD-1 group (*P* < 0.001), and the proportion was also higher in the nsPEF group than in the control group, but this difference was not significant (*P* > 0.05). Consequently, the CD4^+^/CD8^+^ ratio was significantly higher in the nsPEF group than in the anti-PD-1 group (*P* < 0.001) (Figure 2). These results indicated that the immune response triggered by nsPEF ablation was mediated by CD4^+^ T cell infiltration, while anti-PD-1 treatment tended to result in a CD8^+^ T cell response.

### 3.2. NsPEF Ablation and PD-1 Antibody Administration Significantly Increased the Tumor Infiltration of B and NK Cells

**Figure 3:**
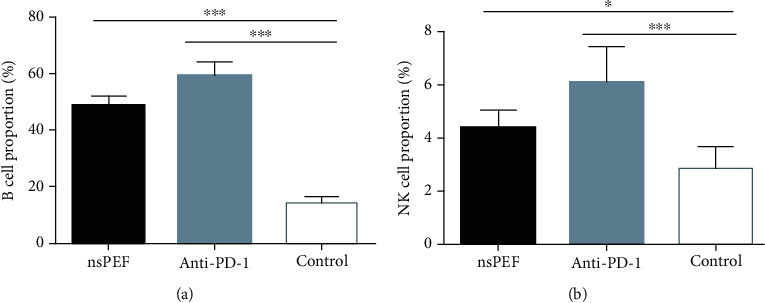
NsPEF ablation and PD-1 antibody administration significantly increased the infiltration of B and NK cells into the tumor. (a) Percentages of B cells within a single-cell suspension of tumor tissues. (b) Percentages of NK cells (values represent means ± SED, ^∗^*P* < 0.05, ^∗∗^*P* < 0.01, ^∗∗∗^*P* < 0.001).

To explore whether humoral immunity and the innate immune response were involved in the treatment responses, we also analyzed the B cell and NK cell populations in tumor tissues. The percentages of B and NK cells were significantly higher in both nsPEF (*P* < 0.001 and *P* < 0.05, respectively) and anti-PD-1 (*P* < 0.001 and *P* < 0.001, respectively) groups than in the control group. However, the magnitude of infiltration was greater in the PD-1 group than in the nsPEF group (Figure 3). These data showed that both methods can enhance humoral and innate immune responses, with a stronger effect of anti-PD-1 therapy.

### 3.3. NsPEF Ablation and PD-1 Antibody Administration Altered Cytokine Concentrations in the Peripheral Blood

**Figure 4:**
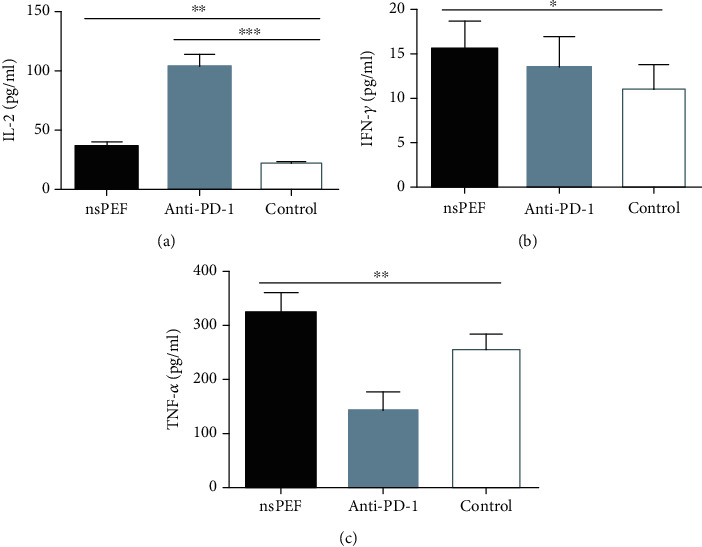
NsPEF ablation and PD-1 antibody administration significantly modulated the concentrations of TH1 cytokines in the peripheral blood. (a) Plasma concentration of IL-2. (b) Plasma concentration of IFN-*γ*. (c) Plasma concentration of TNF-*α* (values represent means ± SED, ^∗^*P* < 0.05, ^∗∗^*P* < 0.01, ^∗∗∗^*P* < 0.001).

**Figure 5:**
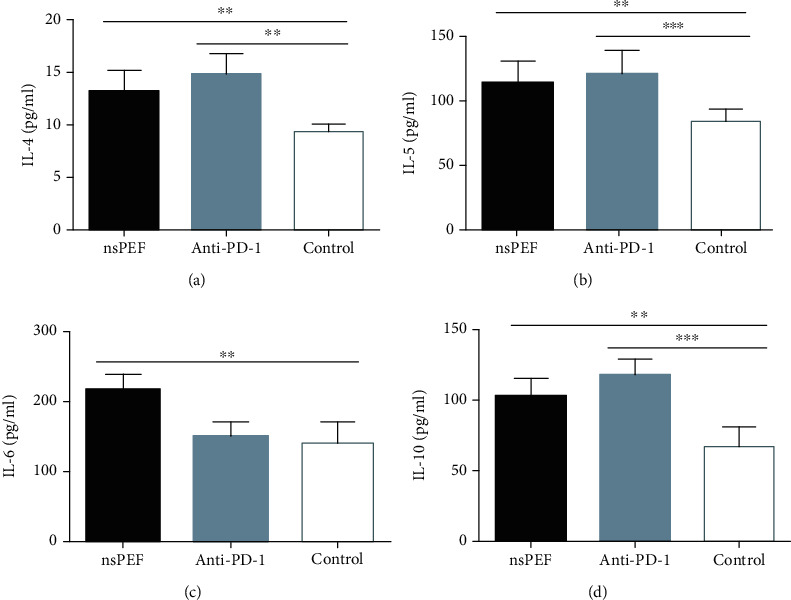
NsPEF ablation and PD-1 antibody administration significantly modulated the concentrations of TH2 cytokines in the peripheral blood. (a) Plasma concentration of IL-4. (b) Plasma concentration of IL-5. (c) Plasma concentration of IL-6. (d) Plasma concentration of IL-10 (values represent means ± SED, ^∗^*P* < 0.05, ^∗∗^*P* < 0.01, ^∗∗∗^*P* < 0.001).

**Figure 6:**
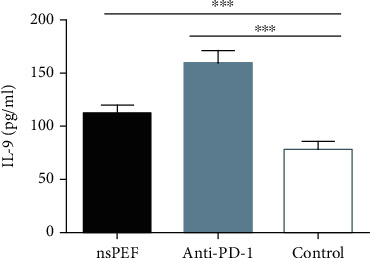
NsPEF ablation and PD-1 antibody administration significantly modulated TH9 cytokine concentrations in the peripheral blood. Plasma concentrations of IL-9 are shown (values represent means ± SED, ^∗^*P* < 0.05, ^∗∗^*P* < 0.01, ^∗∗∗^*P* < 0.001).

**Figure 7:**
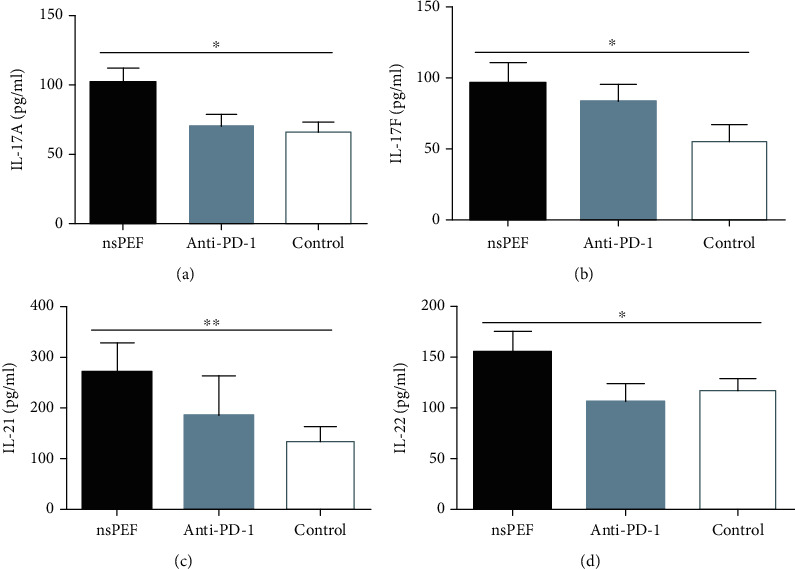
NsPEF ablation and PD-1 antibody administration significantly modulated the concentrations of TH17 cytokines in the peripheral blood. (a) Plasma concentration of IL-17A. (b) Plasma concentration of IL-17F. (c) Plasma concentration of IL-21. (d) Plasma concentration of IL-22.

We evaluated the plasma concentrations of Th1 (IL-2, IFN-*γ*, and TNF-*α*), Th2 (IL-4, IL-5, IL-6, and IL-10), Th9 (IL-9), and Th17 (IL-17A, IL-17F, IL-21, and IL-22) cytokines in tumor-bearing mice by flow cytometry. IL-2, IFN-*γ*, and TNF-*α* concentrations in the nsPEF group were significantly higher than those in the control group (*P* < 0.01, *P* < 0.05, *P* < 0.01, respectively). In the anti-PD-1 group, there was a significant difference in the concentration of IL-2 (*P* < 0.001) but not in IFN-*γ* (*P* > 0.05), and the concentration of TNF-*α* was significantly lower than that in the control group (Figure 4). The concentrations of IL-4, IL-5, IL-6, and IL-10 were also significantly higher in nsPEF group than in the control group (*P* < 0.001, *P* < 0.01, *P* < 0.01, *P* < 0.05, respectively). In the anti-PD-1 group, the differences were significant for IL-4, IL-5, and IL-10 (*P* < 0.001, *P* < 0.001, and *P* < 0.001, respectively), but there was no significant difference for IL6 (*P* > 0.05) (Figure 5). IL-17A, IL-17F, IL-21, and IL-22 concentrations were significantly higher in the nsPEF group (*P* < 0.05, *P* < 0.05, *P* < 0.01, *P* < 0.05, respectively), but differences between the anti-PD-1 group and the control group were not significant (*P* > 0.05) (Figure 6). We also detected significantly higher concentrations of IL-9 in both the nsPEF and anti-PD-1 groups than in the control group (*P* < 0.001) (Figure 7). These results indicate that the two methods triggered a systemic immune response by modulating levels of different cytokines.

## 4. Discussion

Cancer immunotherapy is considered a promising novel approach with numerous advantages, including its ability to not only treat the primary tumor as well as to inhibit tumor recurrence and decrease distant metastases, which still are great challenges in cancer therapy. NsPEF ablation is effective for eliminating early-stage HCC by local ablation and advanced HCC by inducing a remarkable and sustained host immune response. However, the mechanisms by which nsPEF affects the immune status are still unclear. We systematically evaluated the mechanism underlying the activation of local and systemic immune responses by sorting infiltrating lymphocytes and NK cells in local tumors and quantifying cytokine changes in the peripheral blood. Chen et al. have shown that nsPEF ablation in N1-S1 HCC orthotopic model rats yields a response rate of 80–90%; nsPEF eliminates N1-S1 HCC tumors and protects against recurrences for up to 20 weeks by inducing the infiltration of granzyme B-expressing T-cells/NK cells [12]. It has been shown that nsPEF triggers CD8^+^ cytotoxic T-cells, resulting in the inhibition of secondary tumor growth by an immuno-protective, vaccine-like effect [19]. In our study, nsPEF increased the infiltration of T cells, particularly CD4^+^ T lymphocytes the CD4^+^/CD8^+^ ratio was significantly higher than in the anti-PD-1 group, in which tumor-infiltrating cells were predominantly CD8^+^ T cells. The major issues in tumor immunotherapy are immune tolerance and tumor-associated immune suppression. Anti-PD-1 therapy can promote T cell priming and activate preexisting T cells in the tumor [20], while nsPEF can trigger the release of tumor antigens and danger signals, thereby overcoming immune suppression by modulating the TME. In addition to indirect effects, such as CD8^+^ T cell priming and the induction of cytokine production, CD4^+^ T cells can also directly eliminate tumor cells by cytolytic mechanisms via their cytotoxic potential [21, 22]. As already known, the major problem with anti-PD-1 therapy is that patients who lack PD-L1 expression do not benefit from the treatment. It has been indicated that the inflammatory microenvironment induced by RFA treatment plays a vital role in the up-regulation of PD-L1 expression on both tumor cells and tumor-associated immune cells [17]. Therefore, we can estimate that as a locoregional ablation procedure, nsPEF ablation may also up-regulate PD-L1 expression, which will have considerable importance in anti-PD-1 therapy. Accordingly, we can conclude that nsPEF ablation and anti-PD-1 treatment may have complementary effects on the TME and antitumor immune response. We observed significant B cell and NK cell infiltration in both treatment groups. B cells induce humoral immunity by producing multiple antibodies against tumor-associated antigens (TAA), while a high percentage of tumor-infiltrating NK cells is usually associated with a better prognosis for patients with cancer. Furthermore, recent studies have identified an emerging population of cytotoxic, IFN-*γ*-producing innate lymphocyte [23]. These findings in combination with our work indicate that nsPEF ablation may inhibit the evasion of immune surveillance by inducing both innate and adaptive immune responses.

Researchers investigated the cytokine profile of HCC-bearing mice in a time-dependent manner and observed obvious changes in which an unbalanced TH subset returned to a TH1-dominant type after nsPEF ablation [24]. However, in our study, the reversion of cytokine subtypes was not very apparent. Accumulating evidence indicates that Th2 subtype cytokines, which exert immune suppressive effects by inhibiting Th1 cytokine production, are also more potent helpers in B cell activation, and Th2 cytokines can promote B cell proliferation, differentiation, and antibody production, thereby regulating humoral immune responses [25]. From this point of view, it is possible that high B cell levels in both treatment groups may also be maintained by Th2 cytokines. Interestingly, Th17 (IL-17A, IL-17F, IL-21, and IL-22) cytokine concentrations were elevated only in the nsPEF group. It is well known that Th17 cells are critical for tumor progression via their immunosuppressive functions [26]. However, recent studies have indicated that they can also mediate antitumor immune responses by recruiting immune cells into tumors, activating CD8^+^ T cells, or by promoting a reversal toward the Th1 phenotype, and Th17 precursors may produce IFN-*γ* [27–29]. Therefore, Th17 cells probably assist in the maintenance of high IFN-*γ* levels in the nsPEF ablation group.

## 5. Conclusions

In this study, we evaluated the percentage of tumor-infiltrating immune cells and systemic cytokine profile as important indicators of the immune status after nsPEF ablation and PD-1 antibody administration. We observed that the two therapeutic methods activate antitumor immunity by different mechanisms. As a locoregional ablation procedure, nsPEF ablation can trigger potential local and systemic immune responses in HCC-bearing mice. Interestingly, its immune activation effect is partially complementary to that of the PD-1 antibody administration, well-established immunotherapy method. Therefore, we conclude that nsPEF can ablate early-stage HCC and can effectively treat advanced and metastatic HCC by triggering a potential immune response. Additionally, nsPEF combined with PD-1 blockade may be an effective therapeutic strategy, with synergistic antitumor effects in HCC, and this combination will be the focus of our future research. Since nsPEF ablation may up-regulate PD-L1 expression via a direct effect on the inflammatory microenvironment, we plan to investigate the tumor cell PD-L1 expression status after nsPEF ablation, as an indicator of the clinical effect of combined therapy.

## Data Availability

All data generated or analyzed during this study are included in this published article.
